# Application of transurethral prostate resection instrumentation for treating rectal anastomotic stenosis: Case series

**DOI:** 10.1097/MD.0000000000033799

**Published:** 2023-05-12

**Authors:** Wenshan Xu, Yujie Qin, Faying Yang, Jun Qian, Yanbo Dong, Song Tu, Jiaxi Yao

**Affiliations:** a Department of General Surgery II, Hexi University Affiliated Zhangye People’s Hospital, Zhangye Gansu, China; b Department of Endoscopy Center, Hexi University Affiliated Zhangye People’s Hospital, Zhangye Gansu, China; c Department of Urology, Hexi University Affiliated Zhangye People’s Hospital, Gansu, China; d Institute of Urology, Hexi University, Zhangye Gansu, China; e Lanzhou University Second College of Clinical Medicine, Gansu, China.

**Keywords:** prostate resectoscope, radial incision, rectal anastomotic stenosis

## Abstract

Anastomotic stenosis is a common complication of colorectal surgery with anastomosis. To explore a minimally invasive novel approach surgical method for the treatment of rectal anastomotic strictures using transurethral prostate resection instrumentation.

**Methods:**

From 2012 to 2022, 18 patients for the treatment of rectal anastomotic strictures using transurethral prostate resection instrumentation. The transurethral prostate resection instrumentation enters the rectum through the anus to incise the narrow anastomotic orifice in a 4-point radial manner under the resectoscope.

**Results:**

The surgery was successfully completed in 18 patients, and there were no postoperative complications. Postoperatively, 12 patients achieved satisfactory improvement in defecation after 1 incision, and 4 patients underwent another incision 3 months later. Two patients underwent incisions thrice, and the ease of defecation improved in a short period; however, they later underwent permanent colostomy due to repeated stenosis and pain.

**Conclusion:**

The transanal 4-point radial incision of the prostate using transurethral prostate resection instrumentation is a minimally invasive, safe, effective, and simple surgical method for the treatment of rectal anastomotic stenosis supplemented by postoperative dilatation, and is worthy of clinical application.

## 1. Introduction

Benign anastomotic stenosis is a common complication of colorectal surgery.^[1]^ Approximately one-third of anastomotic stenoses originate in the rectum, and many patients undergo surgical resection.^[2]^ With the extensive application of laparoscopy and double staplers in rectal cancer surgery, low anastomosis, and mechanical anastomosis are increasingly common in rectal cancer surgery, and an increasing number of patients with low rectal cancer have retained anal function;^[3]^ however, preserving the anus function results in complications such as anastomotic stenosis, leakage, bleeding, and rectovaginal fistula.

Neoadjuvant radiochemotherapy and anastomotic leakage have been proven to be risk factors for anastomotic stenosis.^[4]^ Therefore, anastomotic stenosis is a common and intractable complication after colorectal tumor surgery with a high incidence.^[5]^ Patients often experience abdominal pain, abdominal distension, difficulty in defecation, and other symptoms that can seriously affect their quality of life. Acute intestinal obstruction can occur in severe cases, which if not handled in time, can be fatal.

Endoscopic interventional therapy, such as balloon dilation, often requires multiple treatments to obtain satisfactory results.^[6]^ However, benign stenosis may have a significant impact on quality of life, including changes in bowel habits, and lead to large intestinal obstructions. After diagnosis and exclusion of local recurrence, the current effective treatment methods are surgery and endoscopy, with endoscopic treatments being the first-line treatment option. The most common endoscopic treatment is expansion.^[7]^ After 1- to 3-fold anastomotic stenosis expansion, many patients can be cured. However, some patients are difficult to cure completely, despite repeated treatments. In clinical practice, most strictures are local and asymptomatic and are found during endoscopy for tumor follow-up. We have performed 4-point radial incisions with a prostate resectoscope through the anus in patients with rectal anastomotic stenosis, with satisfactory results. Herein, we describe the technique in detail.

## 2. Material and methods

### 2.1. General information

Eighteen patients in Hexi University affiliated Zhangye People’s Hospital (11 males and 7 females) between 48 and 74 years of age; 6 were cases of grade I stenosis (anastomotic diameter 10–20 mm), 8 were cases of grade II stenosis (anastomotic diameter 5–10 mm), and 4 were cases of grade III stenosis (2 cases with an anastomotic diameter < 5 mm and 2 cases with complete atresia) (Table 1). Seven patients had anastomotic leakage after surgery for middle and low rectal cancer. The stenosis was considered relieved after the diameter of the anastomotic stoma was > 20 mm following 4-point radial incision and expansion. The length of the stenosis ring varied from 5 to 35 mm. There were 12 cases of membranous stenosis (the length of the stenosis ring was <15 mm) and 6 cases of tubular stenosis (the length of the stenosis ring was >15 mm). The study was officially approved by the Ethics Committee of Hexi University Affiliated Zhangye People’s Hospital and written informed consent was obtained from each patient.

**Table 1:** Clinical characteristics of patients.

Patient	Sex	Age (yr)	Operation to anastomotic stenosis (mo)	Classification	Operation (min)	Bleeding (mL)	Number of stricture incisions (n)	Chemotherapy
1	Male	70	7	II	15	17	1	XELOX
2	Male	61	8	I	36	26	1	FOLFOX
3	Male	73	4	II	45	50	1	XELOX
4	Male	70	5	III	28	27	2	XELOX
5	Male	53	3	II	40	10	1	FOLFOX
6	Female	72	4	I	45	2	1	–
7	Male	44	5	II	36	36	1	–
8	Male	72	6	III	20	43	3*	FOLFOX
9	Female	57	2	I	38	10	1	–
10	Male	52	3	I	40	45	1	FOLFOX
11	Male	58	9	II	36	33	1	XELOX
12	Female	54	5	III	21	5	1	FOLFOX
13	Male	66	5	I	43	28	1	–
14	Female	48	3	III	29	10	3*	–
15	Female	71	13	II	17	0.5	2	FOLFOX
16	Male	74	9	I	46	20	1	–
17	Female	71	11	II	18	39	2	FOLFOX
18	Female	56	4	II	19	37	2	XELOX

FOLFOX = Oxaliplatin + fluorouracil + LV (calcium folinate), XELOX = Oxaliplatin + capecitabine.

* Permanent fistula.

### 2.2. Surgical method

**Figure 1.**
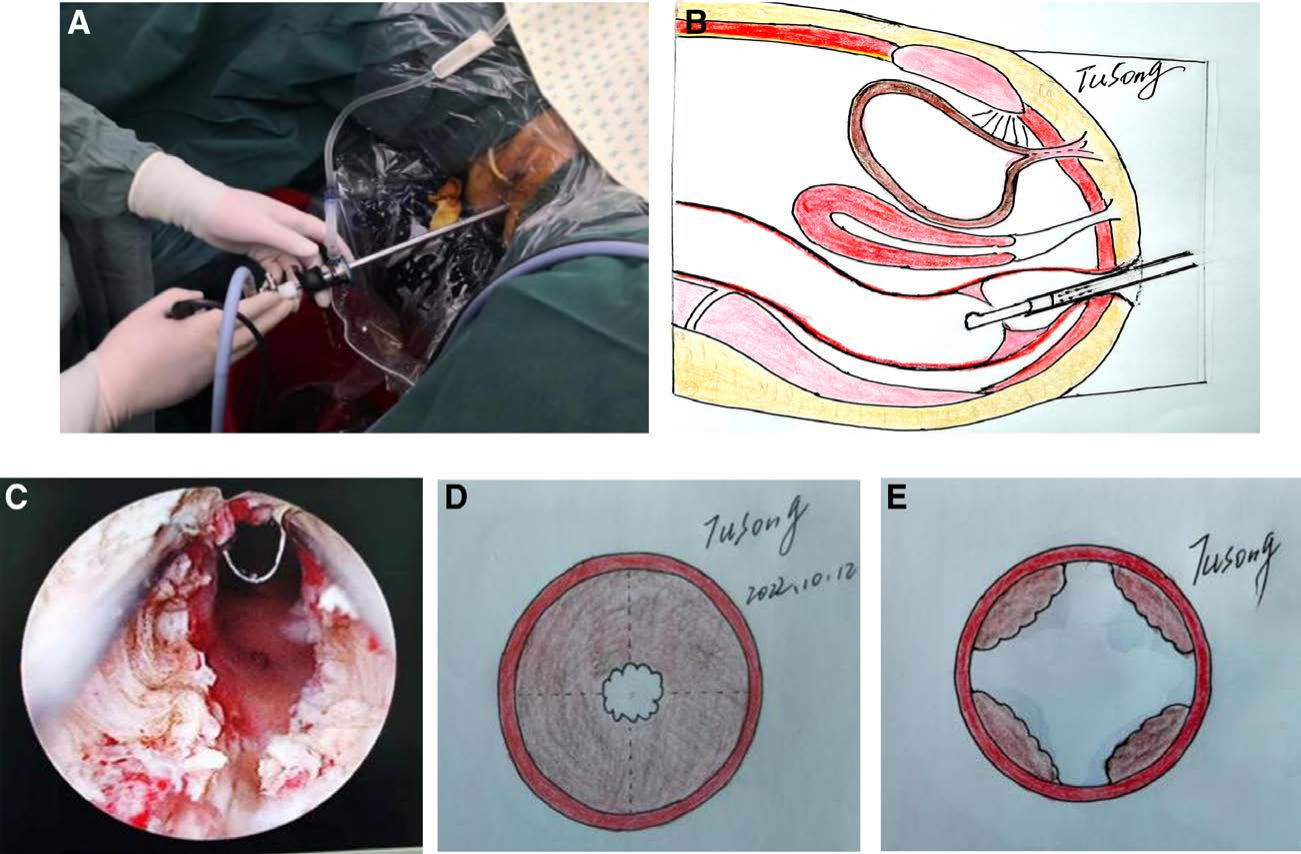
(A) A 26.5-Fr (diameter 8.3 mm) prostate resectoscope inserted into the rectum through the anus. (B) Pattern diagram. (C) Four-point radial incision with transurethral prostate resection instrumentation. (D) Pattern diagram before incision. (E) Pattern diagram after incision.

SIMAI transurethral prostate resection instrumentation with an effective length of 20 cm and diameter of approximately 8.3 mm (Fr-26) was used. A 0.9% sodium chloride solution was used as the flushing fluid, the fluid pressure was set at 40 cm H_2_O, the electric cutting power was set at 280 W, and the electric coagulation power was set at 180 W. The drainage port of the resectoscope was connected to a 30 cm long drainage tube, and the rectal flushing fluid was discharged by siphoning. In the lithotomy position, the patient’s head was maintained 30° higher than the foot to reduce any upward perfusion of the rectal flushing fluid (Fig. 1A and B). The lens was entered into the rectum through the anus to check and evaluate the condition of the rectum and anastomosis (degree of anastomotic stenosis, tumor recurrence, inflammation, bleeding, ulcer, etc) (Fig. 1C). For patients with anastomotic stenosis, 4 straight grooves incisions were cut radially at points 3, 6, 9, and 12 of the lithotomy site under the electric resectoscope (Fig. 1D and E). The depth of the incised stenosis ring reached the surface of the rectal annular muscular layer visible under the transurethral prostate resection instrumentation or the line between the bottom of the cut groove and the rectal mucosa at both ends of the stenosis plane. If fat tissue was observed during the incision, the incision was considered too deep and the operation was terminated immediately. This situation causes easy bleeding; in particular, the presacral region at 6 o’clock and the seminal vesicle of the prostate at 12 o’clock are rich in venous plexuses, and thus venous hemostasis is difficult.

After incision with the help of fingers, the anastomotic stoma was expanded to easily pass 2 fingers (>25 mm in diameter). It was incised and expanded to a satisfactory level, secured hemostasis under the electric resectoscope was carefully stopped, the rectum was washed, and the excised scar tissue of the anastomosis was collected and sent for pathological examination. We retained a catheter above F22 in the rectum for drainage (a 20 mL water-filled airbag was fixed on the narrow ring to prevent it from falling out). For severe anastomotic stenosis that is not large enough to allow the passage of the electric resectoscope, a guidewire can be placed under the guidance of the electric resectoscope, and an inward push incision can be used. When the lens passes through, a forward-pull 4-point radial incision can be made until it is satisfactory. For anastomotic atresia, under the guidance of computed tomography, magnetic resonance imaging, or the guidance of residual rectal staples, an incision can be made from the central tunnel of the atresia. This can then be changed to a 4-point radial incision after opening the tunnel. For patients with tumor recurrence and stenosis, the same method can also be used to relieve rectal obstruction, temporarily improve quality of life, and treat tumor recurrence. Postoperatively, bleeding through the drainage tube may be observed. If there is no bleeding or only a small amount of bleeding, the drainage tube can be pulled after 24 hours. After 1 week, the anal dilator expands the anastomosis to 25 mm, once a week for 6 months. Recheck the rectal endoscopy and evaluate the treatment effect every 3 months.

## 3. Results

The surgery was successfully completed in 18 patients. The operation time was 15 to 46 minutes, and the blood loss was 0.5 to 50 mL. There were no postoperative complications such as bleeding, infection, or water poisoning. After the operation, 12 patients achieved satisfactory improvement in defecation through a single incision, 4 patients received another incision treatment 3 months later (2 patients achieved satisfactory improvement), and 2 patients underwent 3 incisions, and ease of defecation improved in a short time. However, after repeated stenosis, difficulty in expansion, and pain, they underwent a permanent colostomy (Table 1).

## 4. Discussion

Anastomotic stenosis is a common complication after colorectal surgery, with other complications including benign stenosis and malignant stenosis.^[5]^ The latter is usually caused by tumor recurrence. Benign anastomotic stenosis is more common after rectal surgery and has a negative impact on a patient’s quality of life. Patients often have symptoms of incomplete rectal obstruction, such as abdominal pain, abdominal distension, and difficulty in defecation.^[8]^ In severe cases, acute rectal obstruction may occur due to anastomotic atresia. The main cause of benign anastomotic stenosis is hypertrophic scarring, and endoscopic dilation is the primary treatment. Repeated expansion may lead to mucosal damage and increase the incidence of perforation. Endoscopic stent implantation is a better solution for anastomotic stenosis, especially in acute obstruction, as it can quickly relieve symptoms.^[9,10]^ However, the long-term effects of stents are uncertain and stent displacement and restenosis may occur. Anastomotic stenosis can be divided into membranous and tubular types. Truong et al assessed the degree of colorectal anastomotic stenosis as follows: grade I stenosis was 10 to 20 mm in diameter, grade II stenosis was 5 to 10 mm, and grade III stenosis was <5 mm. If the diameter of the anastomotic stoma was >20 mm after expansion, the stenosis was considered to be relieved.^[11]^ This means that invasive treatment is generally not required for anastomotic stomas > 20 mm, while appropriate treatment should be selected for anastomotic stomas < 20 mm to improve the quality of life of patients with minimal trauma.

Several methods have been reported for the treatment of stenosis. Mild stenosis was treated conservatively using observation. The treatment methods for moderate and severe stenosis include finger expansion, dilator expansion, balloon expansion under endoscopy, and incision of the stenosis ring under endoscopy. If the response is poor, stenosis resection and reconstruction of the digestive tract can be performed. Finger anal dilatation is currently the simplest mechanical expansion method and endoscopic treatment is the most widely used method, especially endoscopic stricture ring incision, which can achieve good results.^[12–14]^ Surgical treatments include resection of the stricture through the anus and reconstruction of the digestive tract through laparotomy. At present, there are few reports of resection of strictures through the anus, and the safety and effectiveness of it still need to be supported by large-sample clinical data. Additionally, reconstruction of the digestive tract through laparotomy is extremely difficult and traumatic. It can only be used as the final treatment for patients with perforation and other complications during endoscopic treatment or those with unresponsive stenosis after multiple treatments. In recent years, endoscopic incision has been gradually performed in various endoscopic centers worldwide, with good therapeutic effects and low complication rates.^[5,15–17]^ However, the enteroscope is slender and soft, and the incision knife is more delicate. When making a radial incision, the surgical stability and directivity are poor; hence, it is difficult to accurately master the incision sites and depth.^[18,19]^ If bleeding occurs during the operation, it becomes difficult to maintain a clear vision, affecting the operation or even leading to the termination of the operation. For patients with severe stenosis and atresia, enteroscopy is difficult to perform. In addition, its long learning curve and expensive scalpel hinder its clinical application and promotion.

Since 2012, our team has used transurethral prostate resection instrumentation such as the rectoscope and scalpel, imitating the resection technology of prostate and bladder tumors, to perform 4-point radial incision treatments for cases of rectal anastomotic stenosis. The surgical procedures and main operations are similar to transurethral resection of the prostate and transurethral bladder tumor resection surgery.^[20,21]^ Currently, transurethral resection of the prostate is a classic surgical method for benign prostatic hyperplasia. Transurethral resection of bladder tumors is an important treatment method for nonmuscle-invasive bladder cancers. Almost all medical units in China use this type of equipment and surgical technology. The technical principle of transurethral prostate resection instrumentation is that a radiofrequency electrode transmits high-energy heat to the target tissue, generating intense heat instantly, and causing rapid carbonization of the organic components of the tissue and cells. There is instant evaporation of water from the tissue and cells and rapid decomposition and vaporization of the target tissue. Radiofrequency electrodes can rapidly heat tissue. The temperature of the tissue contact surface exceeds 400°C, which can produce a 2 to 3-mm coagulation layer on the incised tissue. Therefore, it has good coagulation and hemostasis effects. Currently, mechanical staplers are widely used in gastrointestinal surgeries. The circular end-to-end stapler has proven to be particularly suitable for low anastomosis.^[22]^ Stenosis of the anastomotic stoma follows. Non-neoplastic gastrointestinal stenosis is treated conservatively with a bougie, balloon dilation. If conservative measures fail, surgical removal of the stenotic area is necessary. Surgery is accompanied by a risk of perforation, infection, and even death.

Minimally invasive surgery is becoming an important clinical skill. Compared to traditional endoscopic incision, transurethral prostate resection instrumentation has many advantages in the treatment of rectal anastomotic stenosis. First, the overall transurethral prostate resection instrumentation is no longer than 30 cm, which is much lighter than the 150 cm colonoscope. The technique is relatively easier for the operator. Second, the transurethral prostate resection instrumentation body and electric knife are both hard and straight with good directivity, making it easy to accurately determine the cutting depth and location. Third, the transurethral prostate resection instrumentation keeps flushing with flushing liquid; hence, even if bleeding occurs, it is easy to maintain clear vision. Fourth, the transurethral prostate resection instrumentation is thin and hard and can be used as a dilator for severe stenosis. Fifth, basic hospitals in developing countries have transurethral prostate resection instrumentation, and the resectoscope ring is relatively durable and inexpensive, which does not increase medical costs. Sixth, the siphon drainage of the drainage pipe can maintain internal rectal pressure at a safe flushing pressure in contrast to colonoscopy, which requires an air injection, making it difficult to safely control the internal intestinal pressure. In terms of safety, we have formulated a series of plans for possible complications such as water poisoning, intestinal perforation, incision wound bleeding, local infection, and enterogenous sepsis during surgery. These include rectal low-pressure washing, siphon negative pressure rectal drainage, careful cutting above peritoneal reflexes, hemostasis while cutting, head high, foot low, etc. In addition to the incision of anastomotic stenosis, since 2003, our team has also conducted exploratory research on the treatment of rectal anastomotic fistula, rectal cancer obstruction, severe rectal bleeding, early rectal cancer, rectal adenoma, and other diseases using transurethral prostate resection instrumentation^[23,24]^ After nearly 20 years of clinical practice, we have accumulated vast experience without serious complications.

Our study had several limitations. First, It was a retrospective, single-center study. More data and further prospective studies are needed to investigate whether transurethral prostate resection instrumentation is superior to endoscopic procedures for the treatment of rectal anastomotic strictures.

## 5. Conclusion

In this study, 18 patients with rectal anastomotic stenosis were treated with transurethral prostate resection instrumentation, with satisfactory results and no complications. Clinical practice has proven that the incision of rectal anastomotic stenosis under the transurethral prostate resection instrumentation is a minimally invasive, safe, effective, and simple surgical method worthy of clinical promotion and application.

## Acknowledgments

We would like to thank Editage (www.editage.cn) for English language editing.

## Author contributions

**Conceptualization:** Wenshan Xu, Yujie Qin, Song Tu, Jiaxi Yao.

**Data curation:** Wenshan Xu, Yujie Qin, Faying Yang, Yanbo Dong.

**Formal analysis:** Faying Yang.

**Funding acquisition:** Jiaxi Yao.

**Investigation:** Faying Yang, Yanbo Dong.

**Methodology:** Song Tu, Jiaxi Yao.

**Project administration:** Jun Qian.

**Supervision:** Jun Qian.

**Writing – original draft:** Wenshan Xu, Yujie Qin.

**Writing – review & editing:** Song Tu, Jiaxi Yao.
